# Supplementary material for: Modeling glioblastoma heterogeneity as a dynamic network of cell states
Source: Mol Syst Biol. 2021 Sep 16;17(9):e10105. doi: 10.15252/msb.202010105 (PMC8444284; doi:10.15252/msb.202010105)
Supplement: Supplementary file 5 — Source Data for Figure 3 [file MSB-17-e10105-s001.zip › Figure3A_sourcedata/GSEA_3065/nosigdb_state5.GseaPreranked.1622718435914/LARSSON_STATE_2.html]

Details for gene set LARSSON\_STATE\_2[GSEA]

|  || Dataset | state5 |
| Phenotype | NoPhenotypeAvailable |
| Upregulated in class | na\_pos |
| GeneSet | LARSSON\_STATE\_2 |
| Enrichment Score (ES) | 0.9982022 |
| Normalized Enrichment Score (NES) | 3.927504 |
| Nominal p-value | 0.0 |
| FDR q-value | 0.0 |
| FWER p-Value | 0.0 |
Table: GSEA Results Summary

  

Fig 1: Enrichment plot: LARSSON\_STATE\_2      
 Profile of the Running ES Score & Positions of GeneSet Members on the Rank Ordered List

  

| PROBE | GENE SYMBOL | GENE\_TITLE | RANK IN GENE LIST | RANK METRIC SCORE | RUNNING ES | CORE ENRICHMENT || 1 | MALAT1 |  |  | 0 | 1.196 | 0.0213 | Yes |
| 2 | SNTG1 |  |  | 4 | 0.988 | 0.0385 | Yes |
| 3 | VMP1 |  |  | 5 | 0.967 | 0.0557 | Yes |
| 4 | KCNQ1OT1 |  |  | 8 | 0.931 | 0.0721 | Yes |
| 5 | NOVA1 |  |  | 10 | 0.870 | 0.0874 | Yes |
| 6 | PTPRZ1 |  |  | 12 | 0.862 | 0.1027 | Yes |
| 7 | WSB1 |  |  | 13 | 0.861 | 0.1180 | Yes |
| 8 | CD9 |  |  | 14 | 0.857 | 0.1332 | Yes |
| 9 | BCAN |  |  | 15 | 0.850 | 0.1484 | Yes |
| 10 | NEAT1 |  |  | 17 | 0.830 | 0.1630 | Yes |
| 11 | MTRNR2L12 |  |  | 18 | 0.825 | 0.1777 | Yes |
| 12 | PTN |  |  | 19 | 0.824 | 0.1924 | Yes |
| 13 | MDM4 |  |  | 21 | 0.811 | 0.2067 | Yes |
| 14 | MEG3 |  |  | 22 | 0.783 | 0.2206 | Yes |
| 15 | CD63 |  |  | 24 | 0.769 | 0.2342 | Yes |
| 16 | SOX6 |  |  | 28 | 0.754 | 0.2473 | Yes |
| 17 | PDIA6 |  |  | 29 | 0.742 | 0.2605 | Yes |
| 18 | APLP2 |  |  | 31 | 0.733 | 0.2734 | Yes |
| 19 | GRIA2 |  |  | 32 | 0.732 | 0.2864 | Yes |
| 20 | HSP90B1 |  |  | 33 | 0.729 | 0.2994 | Yes |
| 21 | KANSL1 |  |  | 34 | 0.721 | 0.3122 | Yes |
| 22 | PRH1 |  |  | 35 | 0.718 | 0.3250 | Yes |
| 23 | SCRG1 |  |  | 36 | 0.715 | 0.3377 | Yes |
| 24 | PLAT |  |  | 37 | 0.714 | 0.3504 | Yes |
| 25 | APP |  |  | 38 | 0.712 | 0.3631 | Yes |
| 26 | SQLE |  |  | 39 | 0.705 | 0.3757 | Yes |
| 27 | ADGRV1 |  |  | 40 | 0.703 | 0.3882 | Yes |
| 28 | GPM6B |  |  | 41 | 0.702 | 0.4006 | Yes |
| 29 | NKTR |  |  | 42 | 0.700 | 0.4131 | Yes |
| 30 | HSPA5 |  |  | 43 | 0.698 | 0.4255 | Yes |
| 31 | SPCS2 |  |  | 44 | 0.689 | 0.4378 | Yes |
| 32 | TNC |  |  | 45 | 0.680 | 0.4499 | Yes |
| 33 | RCN2 |  |  | 46 | 0.671 | 0.4618 | Yes |
| 34 | FUS |  |  | 47 | 0.665 | 0.4736 | Yes |
| 35 | SF1 |  |  | 48 | 0.655 | 0.4853 | Yes |
| 36 | PDIA3 |  |  | 49 | 0.652 | 0.4969 | Yes |
| 37 | ARGLU1 |  |  | 50 | 0.642 | 0.5083 | Yes |
| 38 | LAPTM4A |  |  | 51 | 0.641 | 0.5197 | Yes |
| 39 | PGRMC1 |  |  | 52 | 0.639 | 0.5311 | Yes |
| 40 | CANX |  |  | 53 | 0.634 | 0.5424 | Yes |
| 41 | COL4A1 |  |  | 54 | 0.634 | 0.5537 | Yes |
| 42 | CALR |  |  | 55 | 0.631 | 0.5649 | Yes |
| 43 | MIR99AHG |  |  | 56 | 0.624 | 0.5760 | Yes |
| 44 | LRPAP1 |  |  | 57 | 0.619 | 0.5870 | Yes |
| 45 | MIAT |  |  | 58 | 0.619 | 0.5980 | Yes |
| 46 | TMEM259 |  |  | 59 | 0.613 | 0.6089 | Yes |
| 47 | GPM6A |  |  | 60 | 0.609 | 0.6197 | Yes |
| 48 | B2M |  |  | 61 | 0.601 | 0.6304 | Yes |
| 49 | ATP6AP2 |  |  | 62 | 0.597 | 0.6411 | Yes |
| 50 | CREBZF |  |  | 63 | 0.596 | 0.6517 | Yes |
| 51 | DDX17 |  |  | 64 | 0.595 | 0.6623 | Yes |
| 52 | LUC7L3 |  |  | 65 | 0.589 | 0.6727 | Yes |
| 53 | SPPL2B |  |  | 66 | 0.587 | 0.6832 | Yes |
| 54 | DDOST |  |  | 67 | 0.586 | 0.6936 | Yes |
| 55 | TMBIM6 |  |  | 68 | 0.584 | 0.7040 | Yes |
| 56 | LAMP2 |  |  | 69 | 0.584 | 0.7144 | Yes |
| 57 | NCAM1 |  |  | 70 | 0.581 | 0.7247 | Yes |
| 58 | DDX39B |  |  | 71 | 0.574 | 0.7349 | Yes |
| 59 | SSR4 |  |  | 72 | 0.573 | 0.7451 | Yes |
| 60 | ZNF75A |  |  | 73 | 0.572 | 0.7553 | Yes |
| 61 | ADGRG1 |  |  | 74 | 0.567 | 0.7654 | Yes |
| 62 | ZNF431 |  |  | 75 | 0.566 | 0.7755 | Yes |
| 63 | SGCE |  |  | 76 | 0.565 | 0.7855 | Yes |
| 64 | ZNF708 |  |  | 77 | 0.564 | 0.7956 | Yes |
| 65 | CALU |  |  | 78 | 0.563 | 0.8056 | Yes |
| 66 | HMGCR |  |  | 79 | 0.562 | 0.8156 | Yes |
| 67 | PRDX4 |  |  | 80 | 0.553 | 0.8254 | Yes |
| 68 | DDR1 |  |  | 81 | 0.551 | 0.8352 | Yes |
| 69 | SLC1A3 |  |  | 82 | 0.550 | 0.8450 | Yes |
| 70 | ANKRD11 |  |  | 83 | 0.550 | 0.8548 | Yes |
| 71 | PON2 |  |  | 84 | 0.546 | 0.8645 | Yes |
| 72 | FGFBP3 |  |  | 85 | 0.545 | 0.8742 | Yes |
| 73 | PCSK7 |  |  | 86 | 0.545 | 0.8839 | Yes |
| 74 | ACSL3 |  |  | 88 | 0.543 | 0.8935 | Yes |
| 75 | LENG8 |  |  | 89 | 0.543 | 0.9031 | Yes |
| 76 | MARCH6 |  |  | 90 | 0.543 | 0.9128 | Yes |
| 77 | HACD3 |  |  | 91 | 0.542 | 0.9224 | Yes |
| 78 | MSMO1 |  |  | 92 | 0.540 | 0.9320 | Yes |
| 79 | COL4A2 |  |  | 93 | 0.540 | 0.9416 | Yes |
| 80 | XRCC2 |  |  | 94 | 0.534 | 0.9511 | Yes |
| 81 | LMAN1 |  |  | 95 | 0.533 | 0.9606 | Yes |
| 82 | LAPTM4B |  |  | 97 | 0.532 | 0.9700 | Yes |
| 83 | GPC1 |  |  | 98 | 0.530 | 0.9794 | Yes |
| 84 | FTX |  |  | 99 | 0.529 | 0.9888 | Yes |
| 85 | TTC14 |  |  | 100 | 0.526 | 0.9982 | Yes |
Table: GSEA details [plain text format]

  

Fig 2: LARSSON\_STATE\_2: Random ES distribution      
 Gene set null distribution of ES for **LARSSON\_STATE\_2**
